# Regional differences in obesity risk, sedentary behavior, and dietary patterns among university students in Saudi Arabia: a multi-region cross-sectional study

**DOI:** 10.3389/fpubh.2026.1980253

**Published:** 2026-10-01

**Authors:** Mohummed H. Alkhraiji, Abdulrahman I. Alaqil

**Affiliations:** 1 Department of Exercise Physiology, College of Sport Sciences and Physical Activity, King Saud University, Riyadh, Saudi Arabia; 2 College of Education, Department of Physical Education, King Faisal University, Al-Ahsa, Saudi Arabia

**Keywords:** dietary patterns, nutrition transition, obesity, overweight, regional variation, Saudi Arabia, sedentary behavior, self-reported BMI

## Abstract

**Background:**

Overweight and obesity are rising among Saudi university students. Guided by Popkin’s nutrition transition theory, this study examined regional variation in overweight/obesity, sedentary behavior, and nutrition-transition dietary patterns.

**Methods:**

This cross-sectional study (March–May 2023) recruited 564 students from four Saudi administrative regions using a multistage sampling approach. Three colleges per university were selected across three academic domains; within each college, one male and one female class per academic level were invited; all eligible students completed the questionnaire. Students with prior type 2 diabetes were excluded. BMI was calculated from self-reported weight and height (overweight/obesity: ≥ 25.0 kg/m^2^). Dietary patterns were identified using principal-component analysis of 17 food groups from a validated 66-item food frequency questionnaire. Physical activity was assessed via IPAQ-SF (sufficient: ≥ 150 min/week); composite sedentary behavior exposure was classified as low (< 8 h/day), moderate (8–12 h/day), or high (> 12 h/day). Logistic regression adjusted for sex, age, income, college type, region, and smoking.

**Results:**

Overweight/obesity prevalence was 17.9% (*n* = 100/558); as BMI relied on self-reported anthropometrics, this is a conservative lower bound. A marked regional gradient was observed: 27.8% of Riyadh students were overweight/obese versus 8.8% in Makkah (*χ*^2^ = 25.48, *p* < 0.001). Region showed the strongest association with overweight/obesity after adjustment: Riyadh (aOR = 4.62, 95% CI 2.23–9.59, *p* < 0.001) and male sex (aOR = 1.96, 95% CI 1.04–3.71, *p* = 0.037) were the only significant predictors. Composite sedentary behavior exposure was high: 82.3% were moderately or highly sedentary (≥ 8 h/day), dominated by mobile phone use (3.75 h/day) and academic study (3.35 h/day). Sedentary behavior was higher in overweight/obese students (15.5 vs. 13.9 h/day; *p* = 0.043). Nutrition-transition dietary patterns were not associated with overweight/obesity (aOR = 0.93, *p* = 0.543).

**Conclusion:**

Region showed the strongest association with overweight/obesity, with Riyadh students at *four-fold* higher risk than Makkah students, suggesting that regional structural and environmental factors may contribute to obesity risk in this population. Prolonged mobile phone use and screen-based sedentarism were prevalent and associated with overweight/obesity. Campus interventions targeting sedentary screen behavior and regional food environments, particularly in Riyadh, are warranted; future studies should employ direct anthropometry to obtain unbiased prevalence estimates.

## 1 Introduction

Overweight and obesity rates have reached pandemic proportions globally: the prevalence of adult obesity has more than doubled since 1990 and affected approximately 16% of adults worldwide by 2022 (1). These trends are responsible for driving the parallel epidemic of non-communicable diseases (NCDs), including type 2 diabetes mellitus (T2DM), cardiovascular disease (CVD), and hypertension, for which excess adiposity is the principal modifiable risk factor (2). In Saudi Arabia, national surveillance data from the Sharik Health Indicators Surveillance System indicate that obesity prevalence has remained stable at 21–22% between 2020 and 2023 across more than 92,000 participants (3), with obesity affecting approximately one in five Saudi adults and nearly two in five overweight (4). A recent systematic review estimated the economic burden of obesity on Saudi Arabia at 6.4 billion US dollars and documented high rates of obesity-related comorbidities including hypertension (67.6%), T2DM (60.7%), and hypercholesterolemia (51.3%) (5).

This trajectory is widely contextualized by Popkin’s nutrition transition theory, which posits that rapid economic development, urbanization, and globalization progressively replace traditional, minimally processed diets with energy-dense, ultra-processed alternatives, while simultaneously reducing occupational and transport-related physical activity in favor of sedentary, screen-based behavior (6, 7). Critically, Popkin and colleagues have argued that as societies advance through this transition, the determinants of obesity may shift from individual dietary behavior toward structural and environmental factors, including the food environment, urban design, and digital technology, that operate at the population level rather than individually (7, 8). Saudi Arabia, one of the world’s most rapidly urbanized and economically transformed high-income economies (9), represents a compelling context for examining whether this structural shift has already occurred. Regional surveillance data suggest it may have: Althumiri et al. identified higher obesity prevalence in more urbanized Saudi administrative regions, suggesting unmeasured environmental exposures, e.g., car dependency, fast-food availability, and reduced walkability, as potential mechanisms (3, 5). These administrative regions differ substantially in urbanization level, economic activity, and infrastructure: Riyadh is the largest commercial and administrative hub, characterized by extensive car dependency and a large fast-food retail sector, while Makkah and the Eastern Province represent distinct urban environments with potentially different food and built-environment exposures (9).

University students represent a strategically critical subpopulation within this transition. Emerging adulthood (18–24 years) is a critical window during which lifelong healthy behaviors are shaped; longitudinal evidence shows that 60.9% of students gain weight during their first year at university, with gains tracking into later adulthood (10). Campus environments are a key driver of this trajectory, shaping dietary choices, physical activity patterns, and sedentary behavior through food availability, timetabling, and digital infrastructure (11). Overweight/obesity prevalence estimates vary widely among Saudi Arabian students according to single-institution cross-sectional studies. These estimates range from approximately 37% at Qassim University (12) to 26.4% in Ha’il (13) to 43.6% (26.5% overweight, 17.1% obese) in the Eastern Province (14), and 40.5% across 11 colleges at Majmaah University (15). These unexplained inter-institutional variations suggest that regional or campus-level contextual factors, rather than individual behavior alone, may be associated with obesity risk for this population. Alongside excess weight, sedentary behavior has emerged as a defining feature of university student life: screen-based sedentarism driven by academic study demands and mobile phone use is endemic, and a meta-analysis of 44 studies found that high screen time was associated with 27% greater odds of overweight/obesity in young people (16). A large, multi-university Saudi survey of 6,975 students found that approximately 58% spent ≥ 7 h per day engaged in sedentary pursuits (17), and a study at King Faisal University documented high rates of physical inactivity across students enrolled in a range of disciplines (18). Despite this evidence, to date no research has examined the regional distribution of overweight/obesity and its correlates, including sedentary behavior and nutrition-transition dietary patterns, across multiple administrative regions of Saudi Arabia based on a theoretically grounded framework. Guided by Popkin’s nutrition transition theory, the current multi-regional cross-sectional study therefore sought to address this gap in the literature in three ways: (i) characterizing the regional distribution of overweight/obesity among Saudi university students; (ii) quantifying the sedentary behavior burden and its association with overweight/obesity in this population; and (iii) examining whether nutrition-transition dietary patterns provide independent explanatory value for overweight/obesity in this population beyond regional and sedentary-behavior factors.

## 2 Methods

### 2.1 Study design and setting

Conducted between March and May 2023, this cross-sectional study followed the Strengthening of the Reporting of Observational Studies in Epidemiology (STROBE) guidelines (19). A self-administered online questionnaire was distributed via Google Forms through institutional email accounts, coordinated by the Deanship of Scientific Research. Participation was voluntary and anonymous; questionnaire completion implied informed consent. Ethical approval was granted by the Institutional Review Board of King Faisal University, Saudi Arabia (KFU-REC-2022-OCT-ETHICS202), and the study was conducted in accordance with the Declaration of Helsinki.

### 2.2 Participants and sampling

The target population comprised students enrolled at Saudi Arabian universities aged > 18. The sample (*N* = 564) included university students across four administrative regions of Saudi Arabia: Makkah (*n* = 220; 39.0%), Eastern Province (*n* = 166; 29.4%), Riyadh (*n* = 161; 28.5%), and other regions (*n* = 17; 3.0%). A multistage sampling approach was used: at the first stage, universities within each region were identified through the Deanship of Scientific Research; at the second stage, three colleges per university were selected covering three academic domains (health and medical sciences, arts and humanities, science and engineering); at the third stage, within each college, one male-only and one female-only class from each of six academic levels (undergraduate years 1–4; master’s and doctoral levels) were identified; at the fourth stage, all students within selected classes were invited to participate. Since participants were nested within institutions and classes, cluster-robust standard errors were applied in all regression models as a sensitivity check. Students with a prior self-reported diagnosis of T2DM were excluded to avoid confounding variables in dietary and anthropometric data. A minimum sample size of 384 was estimated *a priori* based on a 5% margin of error and a 95% confidence level; this threshold was sufficient for overall prevalence estimation but was not derived from a power calculation for between-region comparisons. The primary Riyadh vs. Makkah comparison (*n* = 161 vs. *n* = 220) provided approximately 85% power to detect the observed odds ratio post-hoc. The *n* = 17 ‘other regions’ group was not analyzed independently. The final sample (*N* = 564; males: *n* = 136, 24.1%; females: *n* = 428, 75.9%) exceeded this requirement (20). The female predominance reflects the sex composition of the classes and colleges sampled; all eligible students in selected classes participated (100% response rate). The participating colleges had predominantly female enrolment, consistent with national higher education patterns in Saudi Arabia, where universities operate sex-segregated classes.

### 2.3 Survey instrument

Data were collected using a structured Arabic-language questionnaire comprising four domains: (i) sociodemographic characteristics and anthropometrics; (ii) tobacco use; (iii) physical activity and sedentary behavior; and (iv) dietary intake. The dietary component featured a semi-quantitative food frequency questionnaire (SFFQ) previously adapted and validated for use in Saudi Arabian populations (20, 21). The SFFQ comprised 66 food and beverage items (53 primary items and 13 sub-items). Responses were presented using an 8-point Likert scale Never to More than three times per day; beverages were assessed on a daily cups-or-glasses scale. Each submission was linked to a unique institutional email address to prevent duplicate entries. Missing data were minimal (< 2% for any single variable); participants with incomplete anthropometric data (*n* = 6) were excluded from BMI analyses, and those with incomplete covariate data were excluded listwise from regression models (*n* = 7), yielding *n* = 557 for multivariable analyses.

### 2.4 Outcome variable: weight status

Body weight and height were self-reported. BMI was calculated as weight (kg) divided by height squared (m^2^). Self-reported anthropometric data are subject to well-documented systematic biases: individuals commonly under-report body weight and over-report height, particularly women and younger adults, leading to systematic underestimation of BMI and misclassification of overweight and obese individuals as ‘normal weight’ (22, 23). Studies report average underestimation of measured BMI by 0.5–1.5 kg/m^2^ and misclassification of 20–25% of truly overweight or obese individuals as normal weight (22, 23). In this sample, mean BMI was higher in males (23.4 ± 3.9 kg/m^2^) than females (21.8 ± 4.0 kg/m^2^). Overweight and obesity prevalence estimates based on self-reported BMI should therefore be interpreted as conservative lower bounds of the true population values. BMI was classified according to WHO criteria: underweight (< 18.5 kg/m^2^), normal weight (18.5–24.9 kg/m^2^), overweight (25.0–29.9 kg/m^2^), and obese (≥ 30.0 kg/m^2^) (1). Six participants who returned incomplete anthropometric data were excluded from BMI analyses (valid responses: *n* = 558). For the primary analysis, weight status was dichotomized as overweight/obese (BMI ≥ 25.0 kg/m^2^) versus normal/underweight (BMI < 25.0 kg/m^2^); continuous BMI sensitivity analyses confirmed the same regional pattern (see Table 1).

**Table 1:** Multiple linear regression: unstandardized coefficients for continuous BMI (kg/m^2^).

Predictor	B (95% CI)	*p*
Intercept	21.30 (19.69–22.91)	< 0.001
NTDS (per 1-SD)	−0.14 (−0.48 to 0.21)	0.434
Composite sedentary behavior score (per 1-SD)	0.29 (−0.06 to 0.63)	0.102
PA: Insufficient (ref: Sufficient)	0.48 (−0.72 to 1.68)	0.430
Sex: Female (ref: Male)	−0.74 (−1.63 to 0.15)	0.103
Age: ≥ 20y (ref: < 20y)	0.73 (−0.14 to 1.60)	0.099
Marital: Married/Other (ref: Single)	1.41 (−0.02 to 2.83)	0.053
Income: Middle-high (ref: Low)	−0.09 (−0.61 to 0.43)	0.730
Academic level (ordinal 1–6)	−0.07 (−0.47 to 0.34)	0.742
College: Sciences (ref: Humanities)	1.11 (0.11–2.11)	0.030
Region: Riyadh (ref: Makkah)	1.70 (0.72–2.68)	0.001
Region: Eastern (ref: Makkah)	−0.01 (−1.23 to 1.22)	0.994
Region: Other (ref: Makkah)	1.31 (−0.70 to 3.32)	0.200
Current smoker (ref: No)	−0.13 (−1.42 to 1.16)	0.845

B, Unstandardized.

### 2.5 Exposure variables

#### 2.5.1 Nutrition-transition dietary pattern

Dietary exposure was operationalized using an empirical dietary-pattern approach to better capture how foods are consumed in combination compared to individual-nutrient analysis (24, 25). The 66 SFFQ items were consolidated into 17 food groups: fast food; sweets and confectionery; sugar-sweetened beverages; fats and sauces; red and processed meat; refined grains; full-fat dairy; white meat; vegetables; eggs; fish; low-fat dairy; fruit; legumes; whole grains; tea and coffee; and nuts. Dietary patterns were extracted using principal-component analysis (PCA) with varimax rotation applied to the 17 food-group variables. Sampling adequacy was confirmed via the Kaiser–Meyer–Olkin (KMO) statistic and Bartlett’s test of sphericity. The number of components retained was guided by eigenvalues > 1 and the resultant scree plot; food groups with rotated loadings ≥ 0.30 were treated as salient (24). Standardized factor scores were computed for all participants. Guided by Popkin’s nutrition transition theory (6, 7), the pattern characterized by high loadings on fast food, sugar-sweetened beverages, sweets, refined grains, and full-fat dairy, and low loadings on vegetables, fish, legumes, and whole grains was designated the nutrition-transition pattern; its standardized factor score served as the primary dietary exposure. The PCA yielded four components with eigenvalues > 1, together explaining 48.3% of total dietary variance. The KMO statistics were 0.71 (acceptable) and Bartlett’s test was significant (*p* < 0.001), confirming sampling adequacy. The nutrition-transition pattern was the first component retained (eigenvalue = 2.84, variance explained = 16.7%). Factor loadings for all four retained components are presented in Supplementary Table S1. It was acknowledged that PCA-derived dietary patterns are sample-dependent and may not fully represent population-level dietary Westernization; findings should therefore be interpreted within the context of this sample.

#### 2.5.2 Activity-transition behaviors

Physical activity was assessed using items adapted from the short-form International Physical Activity Questionnaire (IPAQ-SF) (26, 27), capturing frequency (days/week) and duration (min/session) of vigorous-intensity activity, moderate-intensity activity, and walking over the preceding 7 days. Ordinal response categories were converted to midpoint values and summed to estimate total weekly activity volume (min/week). Participants were classified as sufficiently active (≥ 150 min/week) or insufficiently active (< 150 min/week) according to the WHO’s physical activity guidelines (28). Given the ordinal-midpoint conversion method applied to IPAQ-SF categories, activity estimates may overestimate true physical activity volume (27) and should be interpreted with caution.

Sedentary behavior was assessed by summing self-reported daily sitting time across six contextual domains: academic work/study, housework, television viewing, mobile phone use, laptop use, and gaming. Ordinal responses were converted to midpoint hours and summed. This composite sedentary behavior exposure score were used to classify participants as having either low (< 8 h/day), moderate (8–12 h/day), or high (> 12 h/day) daily sedentary exposure (29, 30). Domain-specific estimates are reported separately in Table 1 to allow assessment of individual behavior contributions. Since these domains may overlap temporally (e.g., mobile phone use during television viewing), summed estimates represent an upper bound rather than a discrete behavioral measure. As a sensitivity analysis, the two most likely overlapping domains (television viewing and mobile phone use) were combined into a single screen-time domain; results did not differ materially from the primary analysis.

#### 2.5.3 Covariates

Sociodemographic covariates included sex, age group, nationality, marital status, number of children, monthly household income, academic level (undergraduate years 1–4, master’s, PhD), and college type (health sciences or non-health sciences). Income was categorized into six bands (≤ 3,000 to ≥ 23,000 SAR/month). Income was subsequently dichotomized as low (≤ 8,000 SAR/month) or middle-to-high income (> 8,000 SAR/month), using 8,000 SAR/month as a threshold approximating the median household income in Saudi Arabia (20). Current smoking status (yes/no) was also included.

### 2.6 Statistical analysis

Analyses were conducted using IBM’s SPSS Statistics Package (version 28.0) and R (version 4.3.1). Normality of continuous variables was assessed using the Shapiro–Wilk test and Q-Q plots. Outliers for BMI (< 14 or > 45 kg/m^2^) and composite sedentary behavior score (> 3 SD from mean) were checked; no extreme outliers requiring exclusion were identified. Continuous variables were summarized as means ± standard deviations (or medians and interquartile ranges for skewed distributions); categorical variables as frequencies and percentages. Between-group differences by weight status were assessed using independent-samples t-tests and chi-square tests. Given the potential modifying effect of sex on both sedentary behavior and obesity risk, all primary results are reported for the total sample and stratified by sex where sample sizes permitted. Interaction terms (sex × region; sex × composite sedentary behavior exposure) were tested in the primary logistic regression model. Pearson correlations examined bivariate associations among the nutrition-transition pattern score, sedentary time, and BMI. Binary logistic regression was used to examine associations of the nutrition-transition dietary pattern score (per 1-SD increase), physical activity sufficiency, and sedentary time category with overweight/obesity (BMI ≥ 25 kg/m^2^), adjusting for sex, age, marital status, income, academic level, college type, administrative region, and smoking status. Given that 100 outcome events limit reliable estimation to approximately 10 predictors (events-per-variable ≈ 10), the covariate set was kept parsimonious. Adjusted odds ratios (aOR) with 95% confidence intervals (CI) are reported. McFadden pseudo-R^2^ was calculated as a supplementary measure of effect size for the logistic model. Firth-penalized logistic regression was conducted as a sensitivity analysis to address potential model instability due to sparse data. Model discrimination was assessed using the area under the receiver operating characteristic curve (AUC-ROC). A calibration plot was generated to evaluate agreement between predicted and observed probabilities. Cluster-robust standard errors were applied to account for student nesting within institutions. Multiple linear regression with continuous BMI as the outcome served as a sensitivity analysis. Multicollinearity was assessed using variance inflation factors (VIF; threshold: > 5); logistic model fit was evaluated with the Hosmer–Lemeshow test. Statistical significance was defined as two-sided *p* < 0.05.

## 3 Results

Of 564 participants, 75.9% were female, 97.9% were Saudi nationals, and 97.3% were aged under 25. The majority were single (95.4%), enrolled in undergraduate programs (98.0%), and drawn from Makkah (39.0%), the Eastern Province (29.4%), and Riyadh (28.5%), with the remaining 3.0% from other administrative regions. Approximately half were enrolled in sciences/health-related colleges (47.7%) and half in humanities/social sciences colleges (52.0%); two participants could not be classified and were excluded from college-type analyses. Current smoking status was reported by 7.6%. Full sample characteristics are shown in Table 2.

**Table 2:** Sociodemographic and clinical characteristics of the study sample (*N* = 564).

Characteristic	Total *n* (%)/Mean ± SD	Male (*n* = 136)	Female (*n* = 428)
Age			
< 20 years	164 (29.1%)	32 (23.5%)	132 (30.8%)
≥ 20 years	400 (70.9%)	104 (76.5%)	296 (69.2%)
Marital status			
Single, never married	527 (93.4%)	130 (95.6%)	397 (92.8%)
Married/divorced/other	37 (6.6%)	6 (4.4%)	31 (7.2%)
Administrative region			
Riyadh	161 (28.5%)	55 (40.4%)	106 (24.8%)
Makkah	220 (39.0%)	53 (39.0%)	167 (39.0%)
Eastern province	166 (29.4%)	22 (16.2%)	144 (33.6%)
Other‡	17 (3.0%)	6 (4.4%)	11 (2.6%)
College category			
Humanities/social sciences	293 (52.1%)*	52 (38.5%)	241 (56.7%)
Sciences/health sciences	269 (47.9%)*	84 (61.5%)	185 (43.5%)
Current smoker			
Yes	43 (7.6%)	28 (20.6%)	15 (3.5%)
No	521 (92.4%)	108 (79.4%)	413 (96.5%)
BMI category (*n* = 558)†			
Underweight (< 18.5 kg/m^2^)	97 (17.4%)	8 (6.0%)	89 (21.1%)
Normal weight	361 (64.7%)	103 (77.4%)	258 (61.1%)
Overweight (25.0–29.9 kg/m^2^)	73 (13.1%)	16 (12.0%)	57 (13.5%)
Obese (≥ 30.0 kg/m^2^)	27 (4.8%)	8 (6.0%)	19 (4.5%)
Physical activity sufficiency			
Sufficient (≥ 150 min/week)	465 (85.0%)	122 (91.0%)	343 (82.9%)
Insufficient (< 150 min/week)	82 (15.0%)	12 (9.0%)	70 (17.1%)
Composite sedentary behavior category			
Low (< 8 h/day)	100 (17.7%)	32 (23.5%)	68 (15.9%)
Moderate (8–12 h/day)	160 (28.4%)	44 (32.4%)	116 (27.1%)
High (> 12 h/day)	304 (53.9%)	60 (44.1%)	244 (57.0%)
Continuous variables			
BMI (kg/m^2^)† self-reported	22.2 ± 4.0	23.4 ± 3.9	21.8 ± 4.0
Nutrition-transition pattern score	0.00 ± 0.41	0.02 ± 0.49	−0.01 ± 0.39
Composite sedentary behavior score (h/day)§	14.2 ± 7.5	12.9 ± 7.7	14.6 ± 7.3
Total physical activity (min/week)‖	577 (IQR 260–773)	643 (IQR 315–820)	551 (IQR 242–756)

^*^College percentages exclude two unclassifiable participants (*n* = 562). † BMI derived from self-reported weight and height; six participants with incomplete data excluded; estimates represent conservative lower bounds of true prevalence (20, 21). § Sedentary time reflects summed estimates across six domains; interpreted as an upper bound due to potential overlap between simultaneous contexts. ‖ Reported as median (IQR) due to highly skewed distribution. ‡ Other regions (*n* = 17) comprise Al-Madinah, Tabuk, Ha’il, and the Northern Borders; this group was not analyzed independently given the small cell size.

BMI data were available for 558 participants (98.9%). Mean BMI was 22.2 ± 4.0 kg/m^2^; as BMI was derived from self-reported anthropometrics, observed prevalence figures are conservative lower bounds (22, 23). Mean BMI was higher in males (23.4 ± 3.9 kg/m^2^) than in females (21.8 ± 4.0 kg/m^2^; *p* = 0.001). Using the WHO classification, 17.4% were underweight, 64.7% normal weight, 13.1% overweight, and 4.8% obese; the combined overweight/obesity prevalence was 17.9% (*n* = 100). Overweight/obesity prevalence by sex was 17.9% in males (24/134 with valid BMI) and 17.9% in females (76/424 with valid BMI). Regional variation was conspicuous: overweight/obesity was nearly three times more prevalent in Riyadh (27.3%) than in Makkah (8.6%), and intermediate in the Eastern Province (19.9%; *χ*^2^ = 25.48, *p* < 0.001). Students attending science colleges had higher overweight/obesity prevalence than their counterparts in humanities colleges (22.8% vs. 13.5%; *χ*^2^ = 7.49, *p* = 0.006). Bivariate comparisons by weight status are presented in Table 3. Overweight/obesity prevalence across quartiles of the nutrition-transition dietary pattern score is presented in Supplementary Table S3; prevalence did not differ meaningfully across quartiles, consistent with the dietary homogenization hypothesis.

**Table 3:** Bivariate comparison of key variables by weight status, total sample and stratified by sex.

Variable	Total normal/underweight (*n* = 458)	Total OW/Obese (*n* = 100)	*p* (total)	Male normal/UW	Male OW/Ob	Female normal/UW	Female OW/Ob
NTDS, mean (SD)	0.00 (0.41)	0.01 (0.36)	0.849	0.01 (0.50)	−0.05 (0.44)	0.00 (0.40)	0.02 (0.34)
Composite sedentary behavior score, h/day	13.9 (7.1)	15.5 (8.7)	0.043	12.5 (7.4)	14.0 (8.9)	14.2 (6.9)	15.8 (8.7)
Total PA, min/wk. (IQR)	385 (234–695)	358 (210–715)	0.891	651 (316–848)	614 (280–831)	352 (213–665)	321 (196–697)
PA sufficiency			0.575				
Sufficient	390 (85.2%)	84 (84.0%)		112 (92.6%)	10 (83.3%)	278 (81.8%)	74 (84.1%)
Insufficient	68 (14.8%)	16 (16.0%)		9 (7.4%)	2 (16.7%)	59 (17.4%)	14 (15.9%)
Region			< 0.001				
Riyadh	114 (24.9%)	44 (44.0%)		33 (27.3%)	22 (91.7%)	81 (24.0%)	22 (29.3%)
Makkah	198 (43.2%)	19 (19.0%)		50 (41.3%)	3 (12.5%)	148 (43.8%)	16 (21.3%)
Eastern Province	133 (29.0%)	33 (33.0%)		31 (25.6%)	8 (33.3%)	102 (30.2%)	25 (33.3%)
College			0.006				
Humanities	250 (54.6%)	39 (39.0%)		43 (36.4%)	9 (37.5%)	207 (61.7%)	30 (40.0%)
Sciences	207 (45.2%)	61 (61.0%)		75 (63.6%)	15 (62.5%)	132 (38.3%)	46 (61.3%)

OW/Ob = overweight/obese. *p*-values for total sample; sex-stratified comparisons shown descriptively. NTDS, Nutrition-Transition Diet Score; PA, Physical activity; UW, Underweight.

Sedentary behavior was highly prevalent. Mean total daily composite sedentary behavior exposure score was 14.2 ± 7.5 h/day (median 13.2, IQR 9.0–18.5). Using the study’s classification, 53.9% were classified as *highly sedentary* (> 12 h/day) and 82.3% were *moderately or highly sedentary* (≥ 8 h/day). Female students had significantly higher sedentary time than male students (14.6 ± 7.3 vs. 12.9 ± 7.7 h/day; *p* = 0.025). Mobile phone use (3.75 ± 2.27 h/day) and academic study (3.35 ± 2.15 h/day) were the dominant sedentary contexts in both sexes. Domain-specific estimates are presented in Supplementary Table S4. Sedentary behavior was significantly higher in overweight/obese students than normal/underweight students (15.5 ± 8.7 vs. 13.9 ± 7.1 h/day; *t* = 2.03, *p* = 0.043), a pattern that was observed in both males (14.0 ± 8.9 vs. 12.5 ± 7.4 h/day) and females (15.8 ± 8.7 vs. 14.2 ± 6.9 h/day). However, neither sex-specific comparison reached significance individually, likely due to reduced power in subgroups. Sedentary time correlated positively and significantly with the nutrition-transition dietary pattern score (*r* = 0.14, *p* = 0.001), though its bivariate correlation with BMI was marginal (*r* = 0.08, *p* = 0.061).

Most participants were classified as *sufficiently active* (85.0%; ≥ 150 min/week). The nutrition-transition dietary pattern score did not differ between weight-status groups (overweight/obese: −0.02 ± 0.36 vs. normal/underweight: 0.01 ± 0.42; *p* = 0.507) and was not correlated with BMI (*r* = −0.07, *p* = 0.095), indicating that dietary Westernization and physical activity level did not independently discriminate by weight status in this sample.

In the multivariable logistic regression (Table 4; *n* = 557), administrative region was the strongest predictor of overweight/obesity. Compared with Makkah, students in Riyadh were more than *four times* more likely to be overweight/obese (aOR = 4.62, 95% CI 2.23–9.59, *p* < 0.001); the Eastern Province showed a non-significant positive trend (aOR = 2.07, 95% CI 0.89–4.80, *p* = 0.091). Male sex was independently associated with higher odds of overweight/obesity (aOR = 1.96, 95% CI 1.04–3.71, *p* = 0.037). Sedentary time showed a marginal positive trend (aOR per 1-SD: 1.23, 95% CI 0.99–1.55, *p* = 0.067). Neither NTDS (aOR = 0.93, 95% CI 0.73–1.18, *p* = 0.543) nor physical activity sufficiency reached statistical significance. The model fit was acceptable (McFadden pseudo-*R*^2^ = 0.073; Hosmer–Lemeshow *p* = 0.200; all VIF < 3.3). The AUC-ROC was 0.74 (95% CI 0.68–0.80), indicating acceptable discrimination. The calibration plot showed adequate agreement between predicted and observed probabilities. Firth-penalized logistic regression yielded virtually identical estimates (region Riyadh aOR = 4.58, 95% CI 2.21–9.50; male sex aOR = 1.93, 95% CI 1.02–3.66), confirming model stability. Interaction terms for sex × region (*p* = 0.214) and sex × composite sedentary behavior (*p* = 0.381) were not statistically significant. Cluster-robust standard errors did not materially alter any finding. Sensitivity analysis with continuous BMI (Table 1) confirmed these regional patterns: Riyadh (*β* = 1.70, *p* = 0.001) and sciences college enrolment (*β* = 1.11, *p* = 0.030) independently predicted higher BMI, while NTDS and physical activity remained non-significant. Adjusted odds ratios for all predictors are presented visually in Supplementary Figure S2 (forest plot). Overweight/obesity prevalence by region and sex is presented in Supplementary Figure S1 (grouped bar chart).

**Table 4:** Multivariable logistic regression: adjusted odds ratios for overweight/obesity (BMI ≥ 25 kg/m^2^ vs. < 25 kg/m^2^).

Predictor	aOR (95% CI)	*p*
Intercept	0.21 (0.07–0.62)	0.005
NTDS (per 1-SD)	0.93 (0.73–1.18)	0.543
Composite sedentary behavior score (per 1-SD)	1.23 (0.99–1.55)	0.067
PA: Insufficient (ref: Sufficient)	1.18 (0.62–2.26)	0.614
Sex: Female (ref: Male)	0.51 (0.27–0.96)	0.037
Age: ≥ 20y (ref: < 20y)	1.18 (0.66–2.12)	0.574
Marital: Married/Other (ref: Single)	2.11 (0.87–5.15)	0.099
Income: Middle-high (ref: Low)	0.96 (0.62–1.48)	0.850
Academic level (ordinal 1–6)	0.92 (0.70–1.21)	0.571
College: Sciences (ref: Humanities)	1.52 (0.81–2.85)	0.196
Region: Riyadh (ref: Makkah)	4.62 (2.23–9.59)	< 0.001
Region: Eastern (ref: Makkah)	2.07 (0.89–4.80)	0.091
Region: Other (ref: Makkah)	2.54 (0.71–9.07)	0.151
Current smoker (ref: No)	1.26 (0.56–2.81)	0.573

aOR, Adjusted odds ratio; CI, Confidence interval. Model: *N* = 557, McFadden pseudo-*R*^2^ = 0.073, likelihood-ratio *χ*^2^ (14) = 38.19, *p* < 0.001; Hosmer–Lemeshow *χ*^2^ = 11.03, *p* = 0.200; all VIF < 3.3.

## 4 Discussion

This multi-regional cross-sectional study examined the regional distribution of overweight/obesity, the burden of sedentary behavior, and the contribution of nutrition-transition dietary patterns to weight status among 564 Saudi university students. Two substantive findings emerged. First, administrative region was the dominant independent predictor of overweight/obesity: students in Riyadh carried more than *four-fold* higher adjusted odds than students in Makkah (aOR = 4.62, 95% CI 2.23–9.59, *p* < 0.001), a gradient that persisted after adjusting for sex, income, college type, dietary pattern, and sedentary time. Second, sedentary behavior was near-universal: 87.2% of participants exceeded 7 h of daily sitting and 53.9% were classified as highly sedentary (> 12 h/day); sedentary behavior was significantly higher in overweight/obese students than in their normal or underweight counterparts (15.5 vs. 13.9 h/day, *p* = 0.043). Viewed from the perspective of Popkin’s nutrition transition framework, these findings are consistent with the theory’s prediction that rapid economic development, urbanization, and globalization may progressively shift the determinants of obesity from individual dietary behaviors toward structural and environmental factors (2, 8).

The regional gradient in overweight/obesity among Saudi university students is the headline finding of the current work. A near three-fold difference in crude overweight/obesity prevalence between Riyadh (27.3%) and Makkah (8.6%) cannot be explained by individual behavior alone, particularly when it persists after multivariate adjustment. Riyadh, Saudi Arabia’s largest and most economically developed city, may be characterized by higher car dependency, greater fast-food availability, and more intensive commercial food-marketing exposure than other regions, although these environmental features were not directly measured in this study and remain hypotheses requiring confirmation (2, 8, 9). This aligns with national surveillance documenting that more urbanized Saudi regions face a greater obesity burden (3, 5). The sensitivity analysis replicated this pattern using continuous BMI, with Riyadh students having a 1.70 kg/m^2^ higher BMI than Makkah students (*p* = 0.001). Importantly, since BMI was derived from self-reported weight and height data, absolute overweight/obesity estimates were likely conservative: studies comparing self-reported with measured anthropometrics in similar young-adult populations report systematic underestimation of obesity prevalence by 5–10 percentage points (22, 23). The regional gradient itself, however, is unlikely to be an artifact of this bias unless self-reporting accuracy differed systematically across regions, strengthening confidence that this reflects a true association. To the best of our knowledge, the current work is the first to reveal such a regional gradient in overweight/obesity prevalence, specifically within the Saudi university student population, a finding which remains indiscernible in single-institution designs.

The high composite sedentary behavior exposure burden highlighted in the current results warrants careful interpretation. Total mean composite score was 14.2 h/day, with 82.3% classified as *moderately or highly sedentary* (≥ 8 h/day). Mobile phone use (3.75 h/day) and academic study (3.35 h/day) were the dominant contexts for sedentary behavior in both males and females. For context, one study reported that 58% of 6,975 Saudi university students exceeded 7 h/day of sedentary time (17); the higher proportion documented in the present study likely reflects its multi-domain summing approach, which may double-count concurrent behaviors (e.g., mobile phone use while watching television) and should be interpreted as an upper bound. The high PA sufficiency rate (85.0%) should also be interpreted cautiously as the ordinal-midpoint conversion applied to IPAQ-SF response categories is likely to overestimate true physical activity volume (27). Nevertheless, mobile phone use emerged as the single largest sedentary domain, consistent with international meta-analytic evidence linking smartphone screen time to 27% greater odds of overweight/obesity in young people (16), and with mechanistic evidence that sedentary screen time may displace light-intensity activity and exposes users to food advertising (31). The significant bivariate association between sedentary time and overweight/obesity (*p* = 0.043) and the marginal adjusted trend (aOR = 1.23, *p* = 0.067) are consistent with this evidence base. Notably, the association between sedentary time and overweight/obesity was observed in both male and female students, though neither sex-specific comparison reached statistical significance individually, likely due to reduced subgroup power. Interaction testing found no significant sex × region (*p* = 0.214) or sex × composite sedentary behavior (*p* = 0.381) interactions, indicating that these associations did not differ significantly by sex, though the study was underpowered for formal interaction testing. Saudi Arabia’s national transformation initiative, Vision 2030, sets out physical activity policies that focus largely on increasing moderate-to-vigorous activity; the present findings suggest that sedentary behavior reduction, may warrants equal policy priority in university settings (32).

The absence of a significant association between the nutrition-transition dietary pattern score and overweight/obesity requires interpretation rather than dismissal (24). At least three explanations are plausible. First, in populations where Westernized dietary patterns have become the norm, as appears to be the case with Saudi Arabia, within-sample variation in dietary exposure may be insufficient to detect weight-status differences visible in populations spanning a wider dietary range. This dietary homogenization is itself a hallmark of the advanced state of Saudi Arabia’s nutritional transition (6, 7) in which structural and environmental factors may increasingly be associated with population-level obesity (2, 8). Second, self-reported dietary data from food-frequency questionnaires are vulnerable to misclassification and social-desirability bias, attenuating true associations with adiposity (24). Third, reverse causation cannot be excluded: students who are already overweight may have modified their diets in response. It was also acknowledged that PCA-derived dietary patterns are sample-dependent and may not fully capture population-level dietary Westernization (24). Notably, the nutrition-transition dietary pattern score and sedentary time were positively correlated (*r* = 0.14, *p* = 0.001), indicating that the dietary and activity transitions co-occur at the population level, consistent with Popkin’s framework (6, 7), even when individual dietary variation does not independently predict weight status. Overweight/obesity prevalence did not differ meaningfully across NTDS quartiles (Supplementary Table S3), further supporting the dietary homogenization interpretation.

Male sex was independently associated with higher odds of overweight/obesity (aOR = 1.96, *p* = 0.037), diverging from national Saudi surveillance data which consistently report higher obesity prevalence among women (4, 5). This most plausibly reflects the heavily female-skewed sample composition (75.9% female): if female participants were disproportionately drawn from health-conscious subgroups, female overweight/obesity prevalence would be underestimated. As reported in the Results (Table 1), sciences college enrolment was independently associated with higher BMI (*β* = 1.11, *p* = 0.030). This counterintuitive finding, which was not a primary aim of this study, likely reflects unmeasured confounders specific to health-sciences environments, such as irregular meal schedules during clinical placements or longer sedentary study hours, rather than any causal effect of health education on weight. This finding should be interpreted cautiously given the marginal *p*-value and cross-sectional design.

Five methodological limitations of the current work warrant acknowledgement. First, the multistage sampling design and 100% response rate provide stronger external validity than convenience sampling; however, the sample cannot be considered nationally representative as university students from only four of Saudi Arabia’s 13 administrative regions were recruited. The small ‘other regions’ group (*n* = 17) was not analyzed independently, and the sample size was not derived from a power calculation for between-region comparisons; the main Riyadh vs. Makkah comparison was adequately powered *post hoc*, but the inclusion of smaller regions in the logistic model should be treated as exploratory. Second, although the predominance of female participants (75.9%) reflects the gender composition of the colleges sampled, this limits generalizability to male students; future studies should ensure proportional sex representation at the sampling stage. Third, reliance on self-reported body weight and height data consistently underestimates BMI, particularly in women and young adults, with studies reporting average underestimation of measured BMI by 0.5–1.5 kg/m^2^ and misclassification of 20–25% of overweight or obese individuals as ‘normal weight’ (22, 23). The true overweight/obesity prevalence in this sample therefore likely exceeds 17.9%; to overcome this, future studies should employ direct anthropometric measurement. Fourth, non-Saudi nationals (*n* = 12) and postgraduate students (*n* = 11) were retained in the primary analysis; sensitivity analyses excluding these groups showed no material difference in the main findings, and these groups are not considered a meaningful source of bias. Finally, the multi-domain sedentary time methodology likely inflates absolute estimates through double counting of simultaneous behaviors, and the cross-sectional design precludes causal inference; all associations reported here require confirmation in longitudinal and experimental designs before firm policy recommendations can be made. Despite these limitations, the current study’s key strength lies in its multi-regional systematic design spanning four Saudi administrative regions and multiple universities, which revealed the significant regional variation in overweight/obesity in this population, a finding entirely inaccessible to single-institution research designs.

## 5 Conclusion

In this multi-regional sample of Saudi university students, administrative region showed the strongest association with overweight/obesity, with Riyadh students at *four-fold* higher odds than their Makkah counterparts. Composite sedentary behavior exposure was highly prevalent, with 82.3% of students classified as moderately or highly sedentary. Nutrition-transition dietary patterns did not independently predict weight status, most plausibly reflecting dietary homogenization at an advanced stage of the nutrition transition rather than an absence of dietary risk. As BMI was derived from self-reported weight and height data, the observed overweight/obesity prevalence of 17.9% is a conservative lower-bound estimate; future studies should employ direct anthropometric measurement. Universities in Riyadh should be prioritized for campus food-environment and active-transport interventions. Sedentary screen time, particularly prolonged mobile phone use, should be an explicit target in university health programs consistent with Vision 2030’s objectives. Future studies should employ probability sampling, objective anthropometry, longitudinal designs, and direct measurement of campus environmental exposures to confirm these findings and clarify the mechanisms underlying the regional obesity gradient.

## Data Availability

The raw data supporting the conclusions of this article will be made available by the authors, without undue reservation.
