# Supplementary material for: Regional differences in obesity risk, sedentary behavior, and dietary patterns among university students in Saudi Arabia: a multi-region cross-sectional study
Source: Front Public Health. 2026 Oct 1;14:1980253. doi: 10.3389/fpubh.2026.1980253 (PMC13668806; doi:10.3389/fpubh.2026.1980253)
Supplement: Supplementary file 1 [file Data_Sheet_1.docx]

**Supplementary Material**

**Supplementary Table S1. Factor loadings for all four retained components from principal-component analysis (PCA) with varimax rotation applied to 17 food-group variables (N = 558)**

| Food Group | Component 1 Nutrition-Transition Eigenvalue=2.84 Var=16.7% | Component 2 Traditional Eigenvalue=2.11 Var=12.4% | Component 3 Protein-Rich Eigenvalue=1.62 Var=9.5% | Component 4 Healthy Eigenvalue=1.43 Var=8.4% |
| --- | --- | --- | --- | --- |
| Fast food* | 0.71 | 0.08 | 0.12 | 0.05 |
| Sweets & confectionery* | 0.65 | 0.10 | 0.08 | 0.14 |
| Sugar-sweetened beverages* | 0.63 | 0.04 | 0.15 | 0.09 |
| Refined grains* | 0.58 | 0.18 | 0.22 | 0.03 |
| Full-fat dairy* | 0.52 | 0.14 | 0.31 | 0.11 |
| Fats & sauces* | 0.48 | 0.12 | 0.28 | 0.07 |
| Legumes | 0.09 | 0.68 | 0.11 | 0.14 |
| Whole grains | 0.07 | 0.64 | 0.08 | 0.22 |
| Vegetables | 0.11 | 0.61 | 0.14 | 0.31 |
| Fruit | 0.14 | 0.57 | 0.09 | 0.28 |
| Tea & coffee | 0.08 | 0.51 | 0.18 | 0.12 |
| White meat | 0.19 | 0.14 | 0.71 | 0.08 |
| Red & processed meat | 0.28 | 0.11 | 0.68 | 0.04 |
| Eggs | 0.18 | 0.22 | 0.61 | 0.11 |
| Fish | 0.12 | 0.31 | 0.04 | 0.08 |
| Low-fat dairy | 0.08 | 0.28 | 0.14 | 0.72 |
| Nuts | 0.14 | 0.31 | 0.18 | 0.61 |

* Loadings ≥ |0.30| are treated as salient for Component 1 (the Nutrition-Transition pattern). Total variance explained: 48.3%. KMO = 0.71; Bartlett’s test: χ² = 1842.3, p < 0.001. Component 1 is the primary dietary exposure in all regression models.

**Supplementary Table S2. Overweight/obesity prevalence across quartiles of the Nutrition-Transition Dietary Pattern Score (NTDS)**

| NTDS Quartile | Score Range | n | OW/Ob n (%) | p (trend) |
| --- | --- | --- | --- | --- |
| Q1 (lowest) | −1.50 to −0.21 | 140 | 23 (16.4%) | 0.612 |
| Q2 | −0.20 to 0.00 | 140 | 25 (17.9%) |  |
| Q3 | 0.01 to 0.21 | 140 | 26 (18.6%) |  |
| Q4 (highest) | 0.22 to 1.80 | 138 | 26 (18.7%) |  |

OW/Ob = overweight/obese; NTDS = Nutrition-Transition Diet Score. p (trend) from Cochran–Armitage trend test. Overweight/obesity prevalence does not increase meaningfully across NTDS quartiles, consistent with the dietary homogenization interpretation.

**Supplementary Table S3. Individual food group mean intake frequency by weight status**

| Food Group | Normal/Underweight (n=458) Mean (SD) times/week | Overweight/Obese (n=100) Mean (SD) times/week | p |
| --- | --- | --- | --- |
| Fast food | 2.31 (1.89) | 2.48 (2.04) | 0.412 |
| Sugar-sweetened beverages | 3.14 (2.31) | 3.29 (2.44) | 0.561 |
| Sweets & confectionery | 2.18 (1.74) | 2.31 (1.83) | 0.494 |
| Refined grains | 5.41 (2.67) | 5.58 (2.71) | 0.572 |
| Full-fat dairy | 3.62 (2.44) | 3.74 (2.51) | 0.648 |
| Fruit | 3.02 (2.18) | 2.89 (2.09) | 0.614 |
| Vegetables | 4.45 (2.89) | 4.31 (2.74) | 0.672 |
| Fish | 1.24 (1.31) | 1.19 (1.28) | 0.740 |
| Legumes | 2.17 (1.92) | 2.09 (1.88) | 0.715 |

No significant differences in individual food group intake were observed between weight-status groups, consistent with the dietary homogenization interpretation. p-values from independent-samples t-tests.

**Supplementary Table S4. Domain-specific composite sedentary behavior exposure, total sample and by sex**

| Sedentary Domain | Total Mean (SD) h/day | Male Mean (SD) h/day | Female Mean (SD) h/day | Sex difference p |
| --- | --- | --- | --- | --- |
| Mobile phone use | 3.75 (2.27) | 3.42 (2.18) | 3.86 (2.29) | 0.063 |
| Academic study | 3.35 (2.15) | 3.28 (2.19) | 3.37 (2.14) | 0.682 |
| Housework | 2.45 (1.95) | 1.62 (1.71) | 2.71 (1.97) | < 0.001 |
| Laptop use | 1.97 (2.18) | 2.15 (2.31) | 1.91 (2.13) | 0.244 |
| Television viewing | 1.51 (1.83) | 1.55 (1.89) | 1.50 (1.81) | 0.789 |
| Gaming | 1.15 (1.86) | 2.91 (2.54) | 0.61 (0.98) | < 0.001 |
| Total composite score | 14.2 (7.5) | 12.9 (7.7) | 14.6 (7.3) | 0.025 |

Domain-specific scores are presented separately to allow assessment of individual behaviour contributions. The total composite score should be interpreted as an upper bound due to potential temporal overlap between domains (e.g., mobile phone use during television viewing). Significant sex differences were observed for gaming and housework domains. All p-values from independent-samples t-tests.

**Figure S1.** Overweight/obesity (OW/Ob; BMI ≥ 25.0 kg/m²) prevalence by administrative region and sex among Saudi university students (N = 558 with valid BMI data). BMI was derived from self-reported weight and height; all prevalence estimates represent conservative lower bounds of the true population values [20,21]. Three bars per region represent male students (blue), female students (orange), and the total sample combined (grey). The significance bracket indicates the difference in total overweight/obesity prevalence between Riyadh and Makkah (χ² = 25.48, p < 0.001). The ‘other regions’ group (n = 17) is not displayed due to small cell size. Sex × region interaction: p = 0.214. OW/Ob = overweight or obese; BMI = body mass index.

**Figure S2.** Forest plot of adjusted odds ratios (aOR) with 95% confidence intervals (CI) for all predictors of overweight/obesity (BMI ≥ 25.0 kg/m²) from multivariable binary logistic regression (N = 557). Points represent point estimates; horizontal lines represent 95% CIs. The vertical dashed line indicates the null value (aOR = 1.0). Red squares indicate statistically significant associations (p < 0.05); orange squares indicate marginal associations (0.05 ≤ p < 0.10, †); grey squares indicate non-significant associations (p ≥ 0.10). The x-axis uses a log scale. Firth penalized logistic regression confirmed all key findings (Riyadh aOR = 4.58, 95% CI 2.21–9.50; male sex aOR = 1.93, 95% CI 1.02–3.66). Model: N = 557; McFadden pseudo-R² = 0.073; AUC-ROC = 0.74 (95% CI 0.68–0.80); Hosmer–Lemeshow test p = 0.200; all VIF < 3.3. Cluster-robust standard errors applied; no material change to estimates. Reference categories: Makkah (region); Male (sex); Single (marital status); Low income; Humanities (college type); Sufficient physical activity; Age < 20 years. † Marginal association. NTDS = Nutrition-Transition Diet Score; PA = physical activity; SD = standard deviation; HL = Hosmer–Lemeshow; Hum. = Humanities; Sed. = composite sedentary behavior exposure *score.*
